# Extreme conditions research using the large-volume press at the P61B endstation, PETRA III

**DOI:** 10.1107/S1600577522001047

**Published:** 2022-02-22

**Authors:** Robert Farla, Shrikant Bhat, Stefan Sonntag, Artem Chanyshev, Shuailing Ma, Takayuki Ishii, Zhaodong Liu, Adrien Néri, Norimasa Nishiyama, Guilherme Abreu Faria, Thomas Wroblewski, Horst Schulte-Schrepping, Wolfgang Drube, Oliver Seeck, Tomoo Katsura

**Affiliations:** a Deutsches Elektronen-Synchrotron DESY, Notkestraße 85, 22607 Hamburg, Germany; b Bayerisches Geoinstitut, University of Bayreuth, Universitätsstraße 30, 95447 Bayreuth, Germany; c State Key Laboratory of Superhard Materials, Jilin University, Changchun 130012, People’s Republic of China; d Center for High Pressure Science and Technology Advanced Research, Beijing 100094, People’s Republic of China; e Advanced Materials Laboratory, Sumitomo Electric Industries Ltd, 1-1-1 Hyogo, Koyakita 664-0016, Japan; f Helmholtz-Zentrum Hereon, Max-Planck-Straße 1, 21502 Geesthacht, Germany

**Keywords:** extreme conditions, high-pressure, large-volume press, energy-dispersive X-ray diffraction, radiography, resistive heating, ultrasonic interferometry, acoustic emissions detection

## Abstract

Penetrating, high-energy synchrotron X-rays are in strong demand, particularly for high-pressure research in physics, chemistry and geosciences, and for materials engineering research under less extreme conditions. A new high-energy wiggler beamline P61 has been constructed to meet this need at PETRA III in Hamburg, Germany. The first part of the paper offers an overview of the beamline front-end components and beam characteristics. The second part describes the performance of the instrumentation and the latest developments at the P61B endstation. Particular attention is given to the unprecedented high-energy photon flux delivered by the ten wigglers of the PETRA III storage ring and the challenges faced in harnessing this amount of flux and heat load in the beam. Furthermore, the distinctiveness of the world’s first six-ram Hall-type large-volume press, Aster-15, at a synchrotron facility is described for research with synchrotron X-rays. Additionally, detection schemes, experimental strategies and preliminary data acquired using energy-dispersive X-ray diffraction and radiography techniques are presented.

## Abstract 2

The operation of the P61B endstation large-volume press and optics of P61 are reviewed. The instrumentation at P61B, including the large-volume press, detection systems and data acquisition for *in situ* high-pressure experiments are described.

## 1. Introduction

In the last decades, high-energy beamlines have become increasingly available at intermediate- (3–4 GeV) to high-energy (6–8 GeV) synchrotron facilities across the world (see http://wayforlight.eu and http://lightsources.org). The reason for this increase is the interest to study heavy-element and large objects using highly penetrative X-rays. In particular, the third-generation light source PETRA III has the largest circumference in the world, ideal for generating high photon flux at high X-ray energy and for reducing the beam emittance (Franz *et al.*, 2006 ▸). At PETRA III, the beam emittance is reduced with two arrays of damping wigglers (Bacher *et al.*, 2007 ▸). Ten 2 m-long wigglers in the northern straight section of the storage ring serve as a powerful high-energy source for beamline P61 in the P. P. Ewald hall (Tischer & Pflüger, 2004 ▸; Drube *et al.*, 2016 ▸). This beamline offers continuous-spectrum X-rays as opposed to the high flux generated at discrete harmonics from undulators. P61 is split into two stations, P61A is run by the HZ-Hereon group (formerly Helmholtz–Zentrum Geesthacht) and P61B is run by DESY. Both stations share equal access to the high-energy wiggler beam. The Hereon station P61A, focused on using the white beam to perform engineering materials science experiments with controlled gauge volumes, will be described elsewhere. The focus of this paper is on the DESY-run station P61B equipped with a large-volume press (LVP), financed by the Federal Ministry of Education and Research of Germany (BMBF) to study materials under extreme conditions of high pressures and temperatures using *in situ* X-ray techniques.

The P61B endstation accommodates the need for increasing development and demand for high-pressure research (Liebermann, 2011 ▸; Itié *et al.*, 2015 ▸; McMahon, 2020 ▸). Although some types of research in the LVP can be carried out without synchrotron radiation, many studies ideally require the high-intensity and penetrating power of synchrotron X-rays to probe the sample in real time inside an X-ray absorbing environment (*i.e.* the ‘cell assembly’). For high-pressure studies, energy-dispersive X-ray diffraction (ED-XRD) is well established to determine the properties and structure of materials *in situ* under extreme conditions [see *e.g.* 13IDD at the Advanced Photon Source (APS) (Wang *et al.*, 2009 ▸; Yu, Wang *et al.*, 2019 ▸), former NSLS X17B2 and current APS 6-BM beamlines (Weidner *et al.*, 2010 ▸) in the USA, BL04B1 at SPring-8, Japan (Utsumi *et al.*, 1998 ▸; Katsura *et al.*, 2004 ▸) and PSICHÉ at Soleil, France (King *et al.*, 2016 ▸, 2019 ▸)]. Using a (filtered) white beam, the advantage of ED-XRD over angle-dispersive (AD-) XRD is that diffracted X-rays from a small volume of sample can be collected by a point Ge-detector, excluding reflections from the surrounding material (sample capsule, resistive heater and pressure-transmitting medium). Notwithstanding, beamline ID06 LVP at the European Synchrotron Radiation Facility (ESRF), France (Guignard & Crichton, 2015 ▸), and the 13 ID-D beamline (GSECARS, APS) offer AD-XRD in the LVP using a monochromatic beam from an undulator.

Here, we present the design, performance and capabilities of the LVP P61B endstation operated by DESY. The high-intensity polychromatic X-rays from the P61 damping wigglers permit short acquisition times (down to 10 s) to study fast processes *in situ* under extreme conditions using ED-XRD. P61B also offers user access for LVP experiments without synchrotron X-rays, which is a very attractive option and further allows optimal use of the station even when P61A is in operation (with beam). The 6-ram LVP Aster-15, photon detectors and other detection systems, such as acoustic emissions detection and ultrasonic interferometry, are described in detail. A number of results, including an experiment, are presented to show the performance of the detectors and systems at P61B. The station offers a dedicated sample preparation laboratory with all the required infrastructure (*e.g.* cutting/drilling devices, furnaces, a benchtop X-ray diffractometer and a glovebox).

## 2. Overview of the beamline

**Figure 1:**
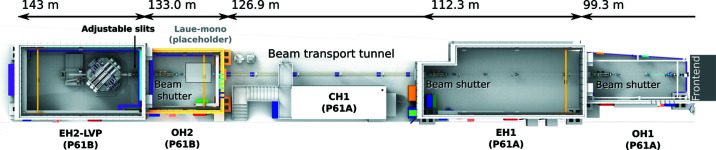
Beamline P61 in sector 1 of the P. P. Ewald hall (PXN) of PETRA III. The distances correspond to the center of the last damping wiggler. The beamline comprises the independently run stations P61A (operated by HZ-Hereon) and P61B LVP (operated by DESY) and share beam time equally. P61B continues to operate the LVP without X-rays for regular users. Both sections of the beamline have a user sample preparation laboratory (not shown). Additionally, P61B runs a dedicated laboratory with a benchtop X-ray diffractometer (CH4).

Beamline P61 is located in sector 1 of the Paul P. Ewald Hall on the north side of the PETRA III storage ring (Fig. 1 ▸). Relevant beam parameters of PETRA III and the ten damping wiggler-array are given in Table 1 ▸. Before delivery to P61, the synchrotron radiation produced by the damping wigglers was blocked by a water-cooled copper absorber unit. To pass through the synchrotron radiation to the new beamline, a special high-power beam absorber was constructed with a rectangular on-axis aperture (3 mm horizontal × 2 mm vertical) and installed behind the last wiggler, replacing the aforementioned copper absorber.

### 2.1. Concrete hutch design

Hutch design generally follows implementations already established for optics hutches and straight sections elsewhere at PETRA III. Calculations for a beam size up to 9 mm^2^ demonstrate the safety requirements of the radiation shielding for the hutch walls and doors (Wroblewski, 2017 ▸). Hence, the P61 hutches are constructed with 300 mm-thick side walls and 500 mm-thick rear walls made of barite-infused concrete. The hutch doors are equipped with heavy 20–25 mm-thick lead plates to absorb the scattered radiation, with the requirement the door can be physically opened. To date, the maximum beam size received at P61B is 4 mm^2^. During beam delivery the doors are locked for radiation safety. The ozone sensor locks the door if the concentration of ozone remains too high after closing the shutter. In this case, the ventilation rate is increased. The door is also locked for safety when the oil pressure in any hydraulic ram of the LVP is higher than 5 MPa.

### 2.2. Optics

**Figure 2:**
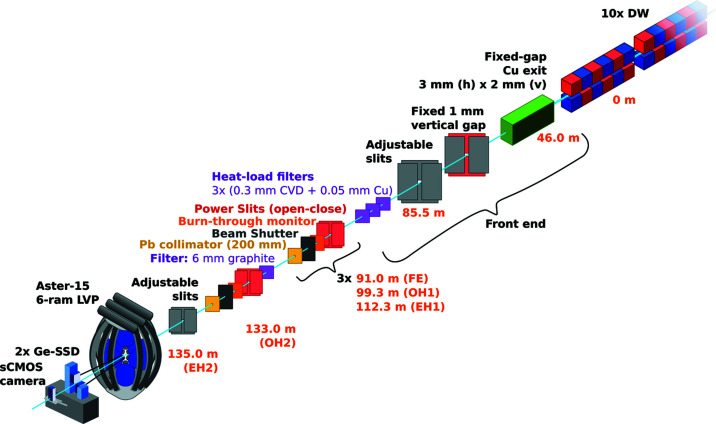
Optical train of beamline P61 with emphasis on the LVP and detector system in the P61B endstation. The optics shown in the brackets are repeated three times in the front end (FE), optics hutch 1 (OH1) and experimental hutch 1 (EH1). In OH2, an additional filter is installed (4 mm graphite). Two slit systems are user-controlled: PS2 in the FE and PS6 in EH2 (P61B). For regular operation, there is always at least one heat-load filter inserted to avoid overheating the beamline components and to reduce the low-energy radiation and production of ozone, both harmful to instrumentation.

**Figure 3:**
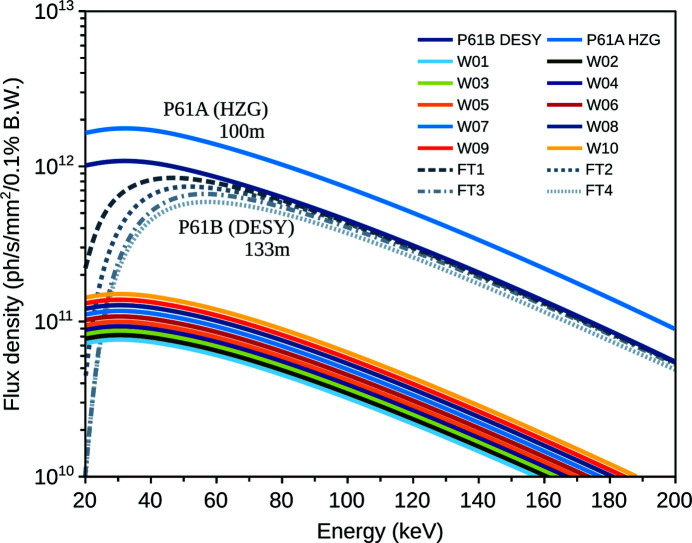
Flux density [photons s^−1^ mm^−2^ (0.1% bandwidth)^−1^] calculated for the damping wigglers of P61 using *SPECTRA* (Version 10.2; Tanaka & Kitamura, 2001 ▸). The individual contributions of each of the ten damping wigglers are shown, as well as the total flux at P61A and P61B (*i.e.* due to the large horizontal beam divergence, at a greater distance, less flux is expected at P61B in a 1 mm^2^ aperture than at P61A). The calculation of the successive contributions of 1 to 4 heat-load filters (FT) are also shown for P61B.

The following devices are installed for the safe operation of the optical elements (Fig. 2 ▸). (1) The vertical beam size is limited to 1 mm using fixed-gap power slits before delivery to the P61 hutches for radiation safety reasons. The horizontal beam size is defined by the alignment of the ten wigglers in the array. (2) In the front end, up to three copper-coated (0.05 mm), CVD diamond (0.3 mm) heat-load filters can be used to prevent over-heating of beamline components and instrumentation (Hahn, 2008 ▸), with at least one such filter required for normal beam operations removing some 25% beam power and a significant amount of flux below 30 keV. The optional two additional front-end filters remove up to 40% beam power at P61B, based on calculations. Indirect observations and dosimetry tests suggest the flux and heat-load calculations using *SPECTRA* (Tanaka & Kitamura, 2001 ▸) (Fig. 3 ▸) are possibly somewhat lower than expected from the wigglers (likely due to the non-perfect alignment of the array). (3) Burn-through monitors are installed for every beam shutter in the event the high-power slits do not function correctly (to absorb greater than allowed beam power and provide an additional layer of protection). (4) High-power slits open and close in front of every beam shutter. The high-power slits remove the heat-load from the beam and block the gas bremsstrahlung from the storage ring, whereas the beam shutter blocks synchrotron radiation up to the highest-energy X-rays. (5) 200 mm lead collimator blocks are installed after every shutter to reduce the spread and scattering of the beam. (7) Retractable diamond beam-position monitors are installed in various locations on the beamline (front-end, OH1, EH1, OH2). Note, all beamline components installed by DESY are in-vacuum and water-cooled.

In addition, a few more components are installed at P61B for LVP experiments. One is a 4 mm-thick glassy carbon filter in the optics hutch (OH2) for imaging-only experiments using X-ray radiography. Adjustable in-vacuum slits (10 mm W-alloy) are also installed for shaping the incident beam for XRD or imaging in front of the LVP in the experimental hutch (EH2). Finally, an in-vacuum, water-cooled Si(111) Laue monochromator is planned, which is currently under design in-house. More information on this component for AD-XRD experiments in the LVP will be described in a future paper.

## 3. Large-volume multi-anvil press

**Figure 4:**
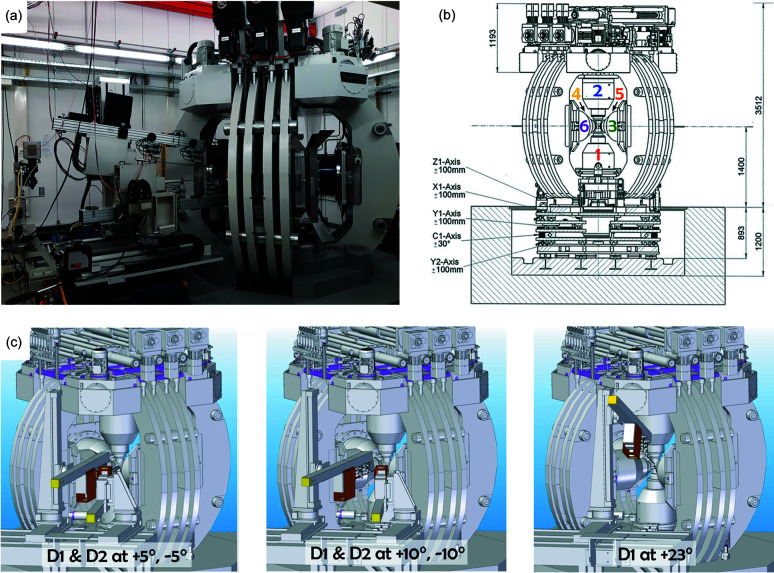
Instrumentation at P61B. (*a*) Photograph of the LVP Aster-15, the detection positioning system with two Ge-SSDs and the X-ray microscope. Note that the plungers for the hydraulic rams are placed on top of the press frame. (*b*) Cross-section schematic of the LVP with the five-movement stages underneath (in a pit). Each stage is labeled accordingly for its movement direction, C1 indicates the rotation stage. (*c*) Various detector arrangements at their limit positions. Note, one Ge-SSD can be at ≥3° as long as the other Ge-SSD is at a position of ≥5° in both horizontal orientations.

The DESY LVP Aster-15 compresses a cubic space with six independent rams controlled by a programmable logic controller (PLC) (Fig. 4 ▸). The maximum load of each axis is 5 MN, equivalent to a uniaxial press with the Osugi-type guide block (Ishii *et al.*, 2019 ▸), with a maximum load of 15 MN in total. This apparatus is a modern version of the Hall-type press (Hall, 1967 ▸). Its prototype was installed at the Bayerisches Geoinstitut (BGI), University of Bayreuth, Germany (Manthilake *et al.*, 2012 ▸). The same type of apparatus was installed at beamline PLANET at the neutron facility J-PARC, Japan (Sano-Furukawa *et al.*, 2014 ▸), and at beamline SAPHiR at the neutron facility FRM-II, Germany. Details of Aster-15 are described below.

### 3.1. Basic design

**Figure 5:**
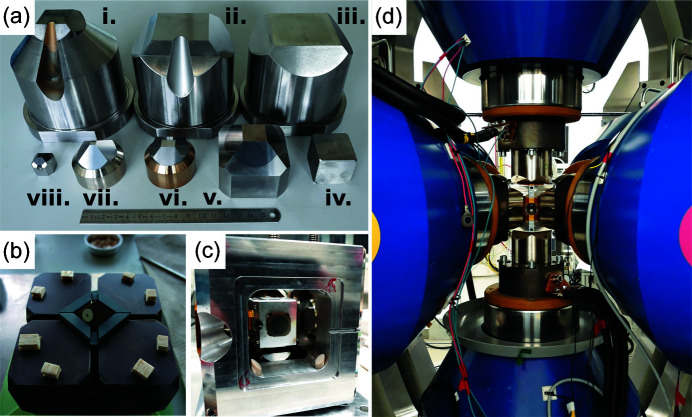
Various anvils and assemblies designed for the LVP. (*a*) First-stage (i, ii and iii), second-stage (iv ‘Kawai’, v, vi and vii ‘cubic’) and third-stage (viii) anvils. (*b*) Half-built assembly for the ‘Kawai’ compression geometry. The octahedral pressure medium with a sample inside is compressed by eight WC cube-shaped anvils. (*c*) Large alignment frame for the cubic compression geometry for WC cylindrical-shaped anvils. Inside the frame, a small alignment frame with smaller third-stage WC/SD anvils (also cylindrical in shape) can be placed. In both geometries, the pressure medium is cubic with a sample inside. (*d*) LVP with the assembly from (*c*) inside, compressed by the large blue hydraulic rams. The conical cuts for the diffracted X-rays and beam exit are clearly visible in the anvils and assembly, respectively.

Three pairs of two rams (six in total) are mounted perpendicular to each other inside a spherical press frame. The six independent plunger pumps, which control the rams individually, are mounted on top of the press frame [Fig. 4 ▸(*a*)]. This design minimizes and fixes the hoses connecting each ram and plunger despite the movement of the press by the alignment stage mentioned later. The pressure of each plunger pump is up to 70 MPa and controlled with a precision of 50 kPa. The absence of guide blocks allows wide openings between the hydraulic rams, permitting vertical and horizontal diffraction angles of 30° and 23°, respectively. The stroke of each ram is 100 mm at the maximum and measured by a displacement gauge with 1 µm precision. The top of each hydraulic ram is socketed for holding a first-stage anvil with various materials, top sizes and conical openings for X-ray transmission (Fig. 5 ▸). Hardened steel first-stage anvils with a top size of 60 mm and a conical opening are most frequently used.

### 3.2. Compression modes

The Aster-15 LVP has six independently acting hydraulic rams. We refer to the bottom and top rams as #1 and #2, respectively. Two pairs of horizontal rams are oppositely mounted, #3–#4 and #5–#6 (Fig. 4 ▸). Note, a pressure profile is always programmed for the master ram (#1 or #3). Aster-15 has three modes of compression. (1) Mode 1: isotropic compression where ram #1 is the master. The strokes of the other rams follow that of #1. This mode is used to generate quasi-hydrostatic pressures in the sample using eight cubic inner anvils with a triangle-shaped truncation and an octahedral pressure medium. This setting is referred to as the 6–8 or ‘Kawai’ mode. (2) Mode 2: anisotropic compression. Ram #3 is the master and rams #1 and #2 (optionally also #5 and #6) can move independently of the master ram to compress the cubic space at a given displacement rate. This mode is used to deform the sample with a near-constant strain rate. Here, smaller second-stage anvils are mounted on each first-stage anvil, referred to as the 6–6 or ‘cubic’ mode. (3) Mode 3: uniaxial compression where ram #1 is the master, and the opposite ram (#2) moves at the same rate as #1. The other rams are deactivated. This mode compresses a squeezer-type assembly for two opposing second-stage anvils, similar to the Paris–Edinburg press.

Because the rams are mounted in the press frame, the apparent displacements given by the stroke gauges on the individual rams are not equal to the displacements of the individual faces of the cubic space. In particular, as a result of frame bending, the displacements of the vertical faces are significantly different from those of the horizontal faces, reaching 1 mm at a near-maximum press load of 60 MPa, because the frame structure is essentially axisymmetric. Hence, the displacements are corrected by the following method. A cube is compressed at incremental steps of 5 MPa to 60 MPa oil pressure in the master ram (similar in the other rams) and recovered after every step. The distances between three pairs of the opposite faces are measured to obtain the deviations from a perfect cube. The deviations are used to fit typically seventh-order polynomials as a function of press load. Corrected displacements are obtained by subtracting these correction values from the measured displacements. However, despite this effort, deviations from a perfect cube more than 50 µm at any high press load are expected, and cannot be further corrected.

### 3.3. Alignment stages

The P61B LVP stands on top of five independent alignment stages [Fig. 4 ▸(*b*)]. The upper three stages, referred to as the *X*1, *Y*1 and *Z*1 axes, linearly translate the sample to the region synchrotron X-rays pass through and from where the diffracted X-rays of the sample are collected by the detector system. Here, *X* is defined as parallel to the incident X-ray beam, *Y* is perpendicular to the incident beam and *Z* is the vertical (up–down) direction. The fourth stage from the top, referred to as the C1 axis, rotates the press around the vertical axis. The lowest stage, referred to as the *Y*2 axis, translates the vertical rotational axis to the incident beam. The translation ranges of the *X*1, *Y*1, *Z*1 and *Y*2 axes are ±100 mm, respectively. The rotational range of the C1 axis is ±15°. More detailed information about the stage functions is summarized in Table S1 of the supporting information. One may wonder why there are two *Y* stages. The primary reason is that the press is too heavy to perfectly place the press rotation center on the synchrotron beam. Another reason is that the rotational axis can be adjusted to the position of a future monochromatic X-ray beam, offset by a monochromator.

### 3.4. Heating systems

Two heating systems are available at P61B. One is a 10 kW DC power supply, and the other is a 3.6 kW AC heating system. The software controlling the AC system is integrated with the press control software, whereas the DC system is independent of it. The software for the press and AC heating system were produced by G. Bauer and S. Linhardt at the BGI. The software for DC heating is designed by us. DC heating allows automatic heating following a pre-programmed profile based on output power. It graphically displays voltage, current, power, resistance with time and, optionally, power against the thermocouple temperature to predict expected output power at thermocouple failure. It supports temperature readouts for up to three thermocouples. The DC system heats the sample with 1 W precision, and is suitable for experiments requiring a maximum pressure up to 20 GPa and temperature up to 2300 K. The AC system controls heating using a Eurotherm thyristor system with the maximum primary voltage and current of 240 V and 15 A with improved sensitivity (steps below 0.1 W) compared with DC heating. The combination of voltage and current on the AC system are transformed to a five-mode step-down transformer for voltage-to-current ratios of 6/600, 10/360, 17/211, 30/120 and 50/72.

Before starting an LVP experiment, one of the two heating systems is chosen based on experimental requirements. If the resistance of a heater is expected to evolve significantly, then DC heating is a better (simpler) choice when no transformer setting on the AC system satisfies any particular voltage and current range. Thermocouple wire is normally insulated from the heater and electrodes inside thin alumina tubes in an assembly. However, contact between these components may occur during compression or the cell design cannot accommodate additional insulation. In these situations, DC current in the heater will negatively affect the thermocouple EMF, whereas AC heating offers a way out as noise from the AC system cancels over one power line cycle. Therefore, the AC system is more suitable for small cell assemblies used to generate very high pressures (>20 GPa). The thick cables of both heating systems can be exchanged on the top and bottom rams of the LVP relatively easily. The cables can also be connected to other portable LVPs.

### 3.5. Pressure calibrations and anvil-cell assembly design

**Figure 6:**
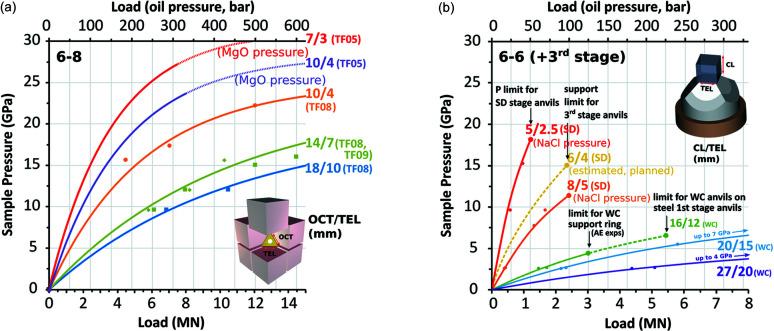
Pressure calibration curves obtained for the various assemblies used in the LVP for (*a*) the ‘Kawai’ 6–8 compression geometry and for (*b*) the cubic 6–6 (+third stage) compression geometry. Note, most pressure curves are only valid at room temperature and do not account for the thermal expansion and stress release and flow of the pressure medium at high temperatures. Typically the entire press load range can be used for 6–8, though this is not the case for 6–6 compression due to the reduced anvil support in the cubic geometry. Some of these limits are indicated in (*b*).

A large variety of cell assemblies have been developed and tested (Fig. 5 ▸). The data points in Fig. 6 ▸ are fixed-point pressure calibrations at room temperature obtained by observing the change in the electrical resistance of Bi (2.55 GPa, 2.7 GPa, 7.7 GPa) (Ono, 2018 ▸) due to phase transitions and the semiconductor-to-metal transitions in ZnTe [9.6 GPa, 12 GPa (Kusaba *et al.*, 1993 ▸)], ZnS [13.4–15.5 GPa (Onodera & Ohtani, 1980 ▸; Ono & Kikegawa, 2018*b* ▸)], GaAs [17.3 GPa (Ono & Kikegawa, 2018*a* ▸)] and GaP [22.2 GPa (Ono & Kikegawa, 2017 ▸)]. A few curves were obtained by X-ray diffraction and the equation of state of MgO or NaCl at room temperature. All curves in Fig. 6 ▸ were obtained by combining hardened steel first stage and commonly available carbide second-stage anvils. Using 6–8 compression, higher pressure can be achieved using very hard carbide anvils with a modified shape (Ishii *et al.*, 2016 ▸, 2017 ▸, 2019 ▸) and using sintered-diamond (SD) anvils (Yamazaki *et al.*, 2014 ▸, 2019 ▸; Yamazaki & Ito, 2020 ▸). Preliminary tests show the Aster-15 LVP can stably generate pressures in experimental assemblies up to at least 50 GPa (Xie *et al.*, 2021 ▸). However, these attempts are not described here because they are too specific for the majority of beamline users. We can generate pressures of 30 GPa and 27 GPa using second-stage anvils with 3 mm and 4 mm truncation, respectively, and TF05-grade carbide supplied by Fujiloy Co. Ltd, whose Vickers hardness is 2200, in the 6–8 mode. We can generate 23 GPa, 17 GPa and 15 GPa using 4 mm, 7 mm and 10 mm truncation, respectively, with carbide of TF08 grade, whose Vickers hardness is 1800, in the 6–8 mode. In the 6–6 mode, we can generate 17 GPa, 15 GPa, 12 GPa, 8 GPa, 7 GPa and 4 GPa using 2.5 mm, 4 mm, 5 mm, 12 mm, 15 mm and 20 mm truncations, respectively.

The following heater materials are typically used. Graphite is used at pressures less than 10 GPa. It can generate temperatures up to 2300 K stably, and is conveniently X-ray transparent. At pressures greater than 10 GPa, LaCrO_3_ and Re are used for experiments without X-rays. Due to the opacity of these materials to X-rays, holes for X-ray windows can be made for these heaters, at a risk of less-stable heating. Skilled experimentalists may orient the cylindrical heater horizontally, in the direction of the X-ray beam axis. Alternatively, other nearly X-ray transparent heaters can be used at high pressures without modifying the assembly significantly, such as TiB_2_+hBN, generating stable temperatures exceeding 1900 K. A new heater material, B-doped diamond, is now routinely used (Nishida *et al.*, 2020 ▸; Xie *et al.*, 2020 ▸). It can generate temperatures over 3500 K and is X-ray transparent.

P61B offers standard user assemblies similar to designs by Sano-Furukawa *et al.* (2014 ▸) in three sizes for the 6–8 mode: 18M, 14M and 10M, where M indicates the octahedral edge length in millimetres. Additionally, large cubic cell assemblies, 27M and 20M, are offered for synthesis experiments without X-rays [Fig. 6 ▸(*b*)]. Since the power–temperature relationships are established for these standard assemblies, no thermocouple is required unless specified. These assemblies cover the total pressure range from <1 GPa to 20 GPa [Fig. 6 ▸(*a*)] without damaging the second-stage anvils in most cases.

Assemblies for rock deformation studies in the 6–6 mode have not been standardized yet, but typically contain hard alumina disks acting as pistons during anisotropic compression. In order to admit all diffracted X-rays to the detector(s), third-stage X-ray transparent SD anvils are used [Figs. 5 ▸(*a*) and 5 ▸(*c*)]. The pressure calibrations in the 6–6 mode are shown in Fig. 6 ▸(*b*). Recent development on a large X-ray transparent cBN anvil has shown promise in enabling low-pressure (0.5–4 GPa) deformation experiments with X-rays on large samples (up to 5 mm). The cBN anvil is compatible with existing WC anvils with truncation edge length of 12 mm and with the setup for acoustic emissions experiments. On the other hand, P61B can offer the anvils for smaller assemblies to users at P61B because of the rarity of *in situ* rock deformation studies.

## 4. Detection systems

The following detection systems are currently available at the P61B endstation.

### 4.1. Standard system

The detection system for synchrotron X-rays at P61B features two high-purity germanium solid-state detectors (Ge-SSD) by Mirion (Canberra) for ED-XRD and a white-beam X-ray microscope by Optique Peter for radiography. In contrast to older generation Ge-SSDs that use a transistor-reset (TSP) preamplifier, limiting count rates to 200 kcps and high dead-time, the Mirion Ge-SSDs are an upgrade featuring a CMOS preamplifier capable of supporting millions of counts per second without saturation, and, under normal conditions, a low dead-time. This capability is significant for P61B due to the extremely high flux (Fig. 3 ▸). The new Ge-SSD supports new features listed in Table S1, as well as a zero-maintenance electric cryostat per detector. We combined the two Ge-SSDs with a single digital analyser, the Quantum detectors Xspress 3 mini, featuring two inputs and 4k channels. The energy–channel relations of the detectors are calibrated using the γ-rays of radionuclides ^57^Co (14.41 and 122.06 keV), ^133^Ba (38.38 keV) and ^241^Am (59 keV) (Fig. S1 of the supporting information). The *K*α and *K*β lines of Mo are also used. The X-ray microscope is combined with a PCO.edge 5.5MP sCMOS camera and two movable objectives (5× and 10× magnification), each fitted with either GGG:Eu or LuAG:Ce scintillators (20–40 µm thickness).

The detectors are positioned on a movable platform with multiple stages designed in-house and constructed by Kohzu, referred to as the detector positioning system (DPS). The main system parameters are summarized in Table S1. A temporary system with a single Ge-SSD and collimator-slit system on stages was used from the first beam in August 2019 to the end of 2020 including the first regular user operation in 2020-II. In the standard configuration [Fig. 4 ▸(*c*)], detector D1 is mounted on translation stages with a goniometer that can rotate vertically from 3° to 23° as well as horizontally from 3° to 10°. For angles below 7.5° in the vertical orientation, a beamstop on a vertical movement stage is necessary before D1 can collect diffracted X-rays from the sample at zero-degree azimuth. Otherwise, the direct beam would hit the detector electronics. In this mode, imaging is only possible once the detector has moved above the beam and the beamstop has moved below the beam. A second detector D2 is mounted on independent translation stages with a horizontal goniometer capable of horizontal rotation (2θ) between 3° and 10°.

In summary, for 6–6 compression, both detectors can be placed (but not necessarily) at the same scattering angle at different azimuthal positions (0° for D1 and 90° for D2). The upper limit of the vertical 2θ range is defined by the press frame, ≤23° for D1 and for the horizontal range to 10° for D2 [Fig. 4 ▸(*c*)]. For 6–8 compression, the horizontal 2θ range is limited to the depth of the conical opening in the first-stage anvils (≤10°) for both D1 and D2, unless the LVP is pre-rotated to accommodate a single Ge-SSD at a position >10°. The minimum angle is 3° for both horizontal positions of D1 and D2 and for the vertical position of D1 (with beamstop).

### 4.2. Scattering angle and gauge volume length

**Figure 7:**
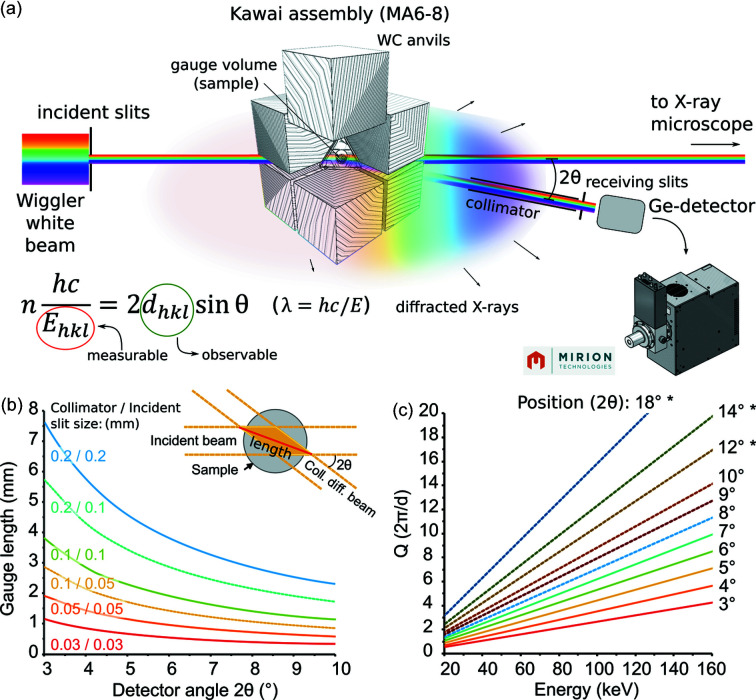
(*a*) Schematic of the principle of ED-XRD based on Bragg’s law for a sample inside an octahedral assembly surrounded by eight WC anvils. (*b*) Calculation of the gauge length (*i.e.* the red line labelled ‘length’) as a function of detector angle position (2θ), incident beam and collimated beam size. (*c*) Calculation of the scattering vector *Q* (Å^−1^) as a function of energy (in the case of ED-XRD) and 2θ (the detector position). The solid lines indicate typically used detector positions and the available *Q* range for these positions up to 160 keV. An asterisk (*) indicates these positions can only be obtained using one Ge-SSD and the LVP pre-rotated to increase the access angle.

P61B offers the user a choice for setting up the ideal detector geometry. The precision and repeatability of the detector positioning system are given in Table S1. For ED-XRD, the following setup is used. A collimator tube, 1350 mm in length with a narrow opening between two pieces of 15 mm WC at the tip, is placed in front of each detector and acts as a scattering slit in the direction of the scattering vector. Before the experiment, one of the following opening sizes can be chosen for the collimator slit: 0.03 mm, 0.05 mm, 0.1 mm or 0.2 mm, where 0.03 mm and 0.05 mm are most commonly used. The distance between the collimator tip and sample is set to ∼200 mm for optimal results compatible with press rotation. Vertical and horizontal receiving slits are located between the collimator and detector. Before the experiment, a choice is made from the following available opening sizes: 0.05 mm, 0.1 mm, 0.2 mm, 0.5 mm, 1.0 mm and 2.0 mm, where 0.2 mm is currently the standard choice for ED-XRD for excellent count rates and a zero-to-low background. The incident beam size, collimator and, to some extent, the receiving slits, define the diffraction angle (2θ) [Fig. 7 ▸(*a*)]. The lengths of the gauge volumes, selected by the detector-collimator-slit system of each detector unit, are calculated as a function of the diffraction angle at various incident-slit and collimator-slit widths [Fig. 7 ▸(*b*)]. The magnitude ranges of the scattering vector are plotted as a function of photon energy at various diffraction angles in Fig. 7 ▸(*c*). The gauge length decreases with increasing diffraction angle, suppressing interfering diffraction from materials in front and behind the sample. On the other hand, the diffraction peaks are more concentrated at the lower energy side, making it difficult to separate each peak. Both incident beam size and collimator slit gap are typically 50 µm. A larger incident beam with a smaller collimator slit gap (*e.g.* 100 µm/50 µm) can be used to gain more intensity owing to the larger gauge volume. The ideal length of the gauge volume is usually 1 mm to 2 mm. When the sample dimensions are smaller, materials that do not interfere with sample diffraction have to be placed in front and behind the sample. Since the diffraction intensity decreases with increasing diffraction angle due to the decrease in the gauge volume, it is better to use a larger collimator slit gap (100 µm, or in extreme cases 200 µm) to receive a sufficient number of diffracted X-rays, or acquisition time should be increased significantly when the diffraction angle is larger than 10° to obtain pair distribution functions of amorphous materials (*e.g.* Yu *et al.*, 2019 ▸).

### 4.3. The acoustic emissions detection system

The acoustic emissions (AE) system at P61B, which can also be used without X-rays, was designed by MISTRAS/GMA (Physical Acoustics Corporation in the USA). It enables investigations of the brittle behavior of materials under pressure, temperature and stress [*e.g.* for understanding the origin of earthquakes (Gasc *et al.*, 2011 ▸, 2017 ▸; Schubnel *et al.*, 2013 ▸; Wang *et al.*, 2017 ▸)]. The system contains three PCI-2 cards with six channels in the frequency range 0.1–3 MHz, with a maximum sampling rate for each channel of 40 MS s^−1^. We offer the micro200HF (MISTRAS) piezo-sensor. In an experiment, each sensor is attached to an anvil of the MA6-6 assembly in the LVP, and connected by a short 300 mm coaxial cable to a 20/40/60 dB gain preamplifier. Six 30 m-long low-attenuation coaxial cables from the preamps transmit the AE signals from the experimental hutch to the data acquisition PC in the control hutch (to avoid X-ray radiation damage to the electronics). The PC system runs the software *AEwin* to acquire AE signals as triggered waveforms and analyses the hit data in real-time for hit time, amplitude, frequency, rise time, duration, energy and counts. If configured, the software calculates the 3D location of events, each formed by six simultaneous hits detected by the sensors on the back of each anvil. Continuous streaming on any channel is also possible, although the total sampling rate on all channels will be lower. The acquired AE data can be replayed and further analyzed after the experiment. A future publication will describe the performance of the AE detection system in more detail.

### 4.4. The ultrasonic interferometry system

A Tektronix AFG3152C arbitrary function generator and Tektronix MSO64 oscilloscope with 1 GHz bandwidth and a sampling rate up to 25 GS s^−1^ were purchased for ultrasonic wave speed measurements for samples at high pressures and temperatures in the LVP (Chantel *et al.*, 2018 ▸; Xu *et al.*, 2018 ▸; Jing *et al.*, 2020 ▸). The ultrasonic interferometry (UI) system is also located in the control hutch. In this case, two of the six available low-attenuation coaxial cables will be used for measurements using a three-way splitter (Mini-Circuits ZFSC-2–1+, 5–500 MHz). One coaxial cable is plugged to the oscilloscope and the other to the waveform generator; they join on the splitter, which is connected via a third (short) coaxial cable to the transducer on the anvil. The transducer is typically chosen to be a thin 10° Y-cut LiNbO_3_ piezoelectric crystal, which can produce both compressional and shear waves. Tests have shown the echoes of the ultrasonic sine waves are dampened in the long cabling (without a preamp), but are still clearly resolvable in part due to the choice of splitter. The hardware of the UI system was chosen for compatibility reasons to be a similar system as at GSECARS, APS, where communication from the PC to the arbitrary function generator and the oscilloscope is scriptable in Python (Jing *et al.*, 2020 ▸). User scripts can be quickly adapted with minor Python knowledge to execute various data collection routines. P61B additionally offers a software tool from the BGI to analyze the acquired waveform data and obtain the two-way travel time for the sample. Combined with the determination of sample length by X-ray radiography, P- and S-wave speeds in the sample can be determined. For a more detailed explanation of the experimental method and choice of sensor, see *e.g.* the work by Jing *et al.* (2020 ▸). Because UI experiments require short travel distances in the WC anvil for the echo of the ultrasonic sine wave, smaller anvils are preferable, 26 mm instead of 32 mm in size. The first-stage anvils in the LVP can be replaced prior to beam time by ones with a top size of 50 mm for these smaller WC anvils. A future publication will describe the performance of the UI system in more detail.

## 5. Experiments and results

Various experimental strategies have been developed at P61B and are described next.

### 5.1. Workflow

**Figure 8:**
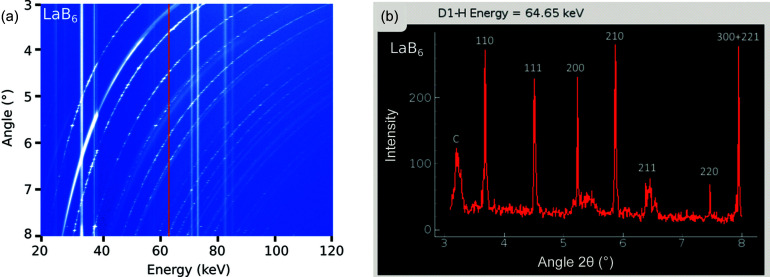
CAESAR data acquisition using the detector system at P61B on LaB_6_ powder inside a graphite cylinder at room pressure and temperature. (*a*) Compiled data from all scans (0.01° increment). (*b*) AD-XRD pattern at 64.65 keV showing seven reflections of LaB_6_ in the range 3° to 8°.

The experimental procedure at P61B is best described by a user guide, as shown in Fig. S2. Note that this chart is applicable to *in situ* studies using X-rays in the LVP, so it does not describe large assemblies available for offline sample synthesis, for instance. The first choice is whether the *in situ* experiment requires application of a deviatoric stress. In most cases, the answer is no, hence 6–8 compression is suitable combined with ED-XRD, currently available with one or two Ge-SSD(s). As mentioned earlier, in the case of ED-XRD, diffraction angles will be chosen for a sample and pressure standard material. Knowledge of the optimal detector position (2θ) and collimator slit gap is useful. An additional mode, referred to as combined angle and energy-dispersive structural analysis and refinement (CAESAR) (Wang *et al.*, 2004 ▸; Itié *et al.*, 2015 ▸), is planned and was recently tested for the first time on the beamline (Fig. 8 ▸). The cell assembly may be modified to optimize the X-ray transmission through the cell components, and B-epoxy X-ray windows can be inserted in the pyrophyllite preformed gaskets on the anvils along the beam direction. The size of the cell assembly and anvil truncation, as well as the heater material and desired heating system (AC or DC heating) are chosen based on the pressure range of interest (Fig. S2).

Following these choices, the cell assemblies for experiments are prepared either at the user laboratory or at the beamline preparation laboratory. The P61B user laboratory is equipped with all the necessary tools and instrumentation for preparation. Instrumentation includes a vacuum oven, storage oven, firing furnace (1000°C), vacuum furnace (1400°C/5 × 10^−5^ mbar), arc welder, low-speed diamond blade saw, polishing wheel, stereomicroscopes, hot-plate, sensitive balance (0.0001 g), hydraulic hand press (15 t) and miscellaneous tools. A user-restricted laboratory is equipped with a three-axis CNC modeling machine, boring machine and lathe for fabricating ceramic components. An MBraun glovebox, funded by the BMBF (PI: Professor Holger Kohlmann), is located in the chemistry laboratory in the same hall. For post-experiment sample analysis, an adjacent laboratory is equipped with a Leica stereomicroscope with a camera and a GNR benchtop X-ray diffractometer (600 W).

Before starting each run, the channel–energy relations of the detector are confirmed using the Pb fluorescence caused by a small number of scattered X-rays from the detector shielding to the Ge-element of the detector due to the imperfect shielding. Pb fluorescence is characterized by *K*α_1_, *K*α_2_ and *K*β_1_ (74.97, 72.80 and 84.94 keV, respectively). If the detector shows disagreement with the observed Pb fluorescence energy, the energy–channel relations should be re-calibrated using radionuclides such as ^57^Co and ^133^Ba. Next, the goniometer angles of one of both Ge-SSDs will be set to the desired diffraction angles. The goniometer angles (*i.e.* detector positions) will be calibrated by taking X-ray diffraction of a reference material (typically MgO) under ambient conditions. Following these steps, the assembly will be placed on the first-stage anvil of ram #1. The sample configuration in the assembly is examined using an X-ray microscope. After locating the sample position on the plane normal to the X-ray incidence by radiography, the pressure-standard is found by scanning the press in the *X* direction and taking diffraction patterns. Once the detectors probe the gauge volume in the pressure standard, a longer-duration XRD acquisition is taken of the pressure-standard under ambient conditions. These data establish the 2θ position of the detector(s) to three or four decimal places.

The sample assembly is compressed to a press load normally less than 1 MN to rediscover the sample- and pressure-marker positions. Although the sample- and pressure-marker positions move by the initial compression, they remain fairly stationary (<0.1 mm displacements) during subsequent compression and decompression.

Note that the very high flux of direct X-rays at P61B have the potential to heat and damage the samples significantly, although all heat load filters combined in the front-end remove a significant portion of the beam power (∼40%). The remaining power (10 W mm^−2^, calculated) is still sufficient to heat samples or the surroundings containing high-*Z* elements dramatically. For example, gold at ambient pressure will heat up and melt near-instantaneously in a large beam (*e.g.* 1 mm^2^). In addition, pre-synthesized meta-stable starting material (*e.g.* bridgmanite) cannot be exposed to the beam until pressurized in the LVP, or the beam will disintegrate or react the sample before the experiment has started. Therefore, if the sample is temperature-sensitive at room pressure, only a narrowly shaped beam can be used, or the sample location has to be determined entirely by XRD stage-scanning.

### 5.2. *In situ* studies of rock deformation

There is growing interest in the mechanical behavior and microstructure/defects of materials, particularly Earth materials, under deviatoric stresses and simultaneously at high pressures and temperatures. Most notably, in the USA the National Science Foundation (NSF) funded a five-year endeavor titled *In situ Studies of Rock Deformation* (ISRD), a research coordination network geared towards the study of candidate Earth materials subjected to deformation under extreme conditions in various LVPs at synchrotron facilities (see https://www.isrdrcn.org/). P61B is involved as an international partner in the advisory committee. Also, in Europe various high-pressure beamlines are active in the field of rock deformation combined with synchrotron X-ray diffraction and imaging [*e.g.* using a modified Paris–Edinburgh press (RoToPEc) at PSICHÉ (Soleil) and at ID27 (ESRF), and a DIA-type press with a deformation module at ID06 LVP (ESRF)].

*In situ* rock deformation experiments at P61B are now possible, also in combination with the AE system. Due to the large sample chamber in the LVP, a new discrete third stage was implemented. Small, X-ray transparent, SD anvils in an alignment frame are compressed by larger second-stage WC anvils inside a larger alignment frame [Fig. 5 ▸(*c*)]. The inner assembly can be independently prepared before placing on top of the second-stage WC anvil. Then the other WC anvils are pushed in, aligned and lightly fixed in place with PEEK rods behind screws [Fig. 5 ▸(*c*)]. The whole assembly is placed inside the press on the first-stage anvil attached to the bottom ram [Fig. 5 ▸(*d*)]. For rudimentary stress estimation during deformation on a sample at high pressure and temperature, the two Ge-SSDs can be positioned and aligned such that both observe the diffracted X-rays from the sample at the same 2θ at two different azimuthal positions (0° and 90°). In this geometry, the expected variation of the lattice *d*-spacings, *d*(*hkl*), of a material as a function of the azimuth follows the relationship (Singh *et al.*, 1998 ▸)

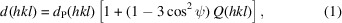

where *d*_P_(*hkl*) is the lattice *d*-spacing at isostatic pressure (zero stress), ψ is the azimuthal angle and *Q*(*hkl*) is the lattice micro-strain parameter. This parameter can be further evaluated to extract the deviatoric stress for each *hkl* using the known material elastic constants and shear modulus at given pressure and temperature conditions. At present, due to the position of D1 above the direct beam, the smallest 2θ is 7.5° to avoid collision between the direct beam and the detector. An actively cooled beamstop is under development and will soon allow collection of diffracted X-rays in D1 at scattering angles >3°.

Future deformation and crystallography studies in the LVP will benefit by using AD-XRD with monochromatic X-rays (see *e.g.* Hilairet *et al.*, 2012 ▸; Farla *et al.*, 2017 ▸) and an area detector.

### 5.3. Beamline user experiments

P61B has been in user operation with synchrotron X-rays since August 2020, receiving 50% of the available beam time. A number of *in situ* studies are already in the press or published, such as: (1) the explanation for the observation of a depressed 660 km discontinuity caused by the akimotoite–bridgmanite transition (Chanyshev *et al.*, 2022 ▸); (2) the determination of phase relations of the Olivine–Ahrensite transition in the Mg_2_SiO_4_–Fe_2_SiO_4_ System at 1740 K and 7.5–11.2 GPa using modern multi-anvil techniques (Chanyshev *et al.*, 2021 ▸); (3) the simultaneous generation of ultra-high pressure and temperature to 50 GPa and 3300 K in multi-anvil apparatus (Xie *et al.*, 2021 ▸); (4) an electrically conductive and ferromagnetic nano-structure manganese mono-boride with high Vickers hardness (Ma *et al.*, 2021 ▸). These studies demonstrate that the beamline is ready to produce publishable data from beam time for an increasing variety of LVP science cases using *in situ* X-ray techniques. The key data obtained using the detection system are described next.

### 5.4. Quality of ED-XRD data

**Figure 9:**
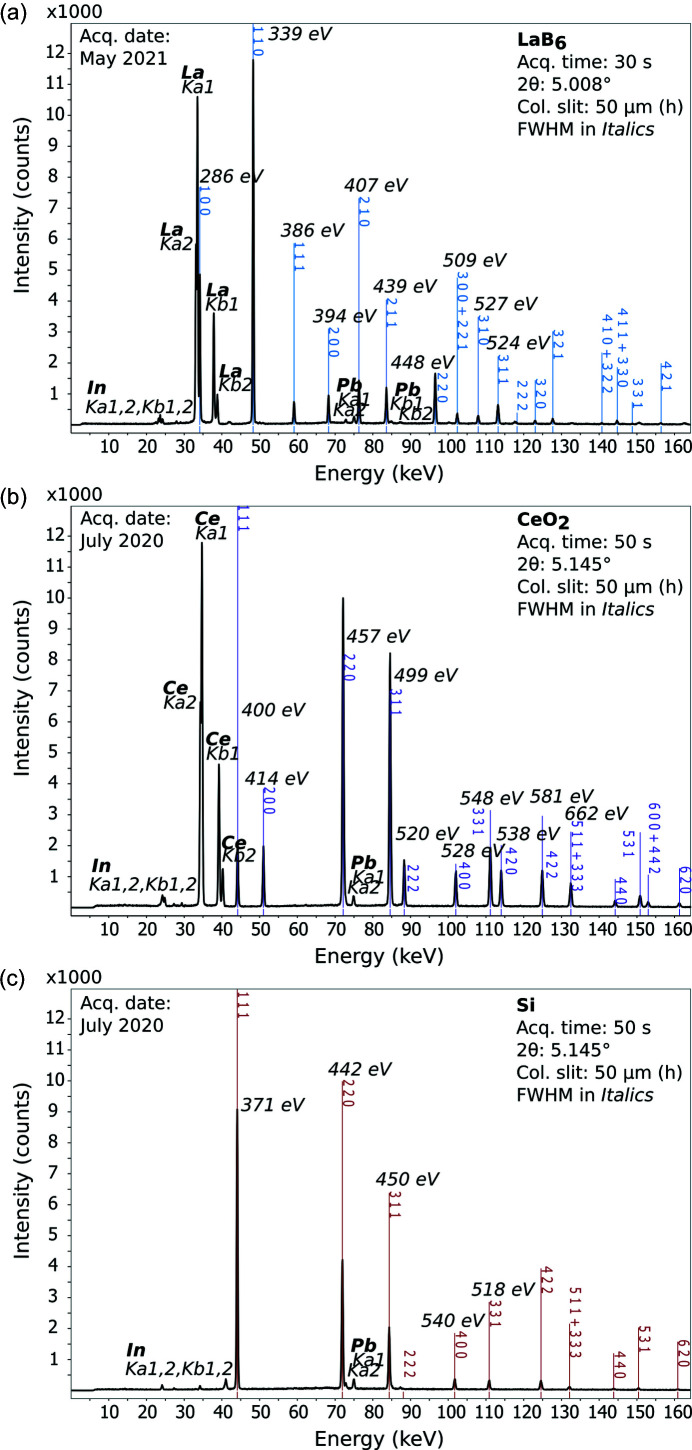
Diffraction patterns obtained from NIST standards: (*a*) LaB_6_, (*b*) CeO_2_ and (*c*) Si. The conditions (acquisition time, detector position, collimator slit gap) are indicated for all patterns. Peak fitting (symmetric pseudo Voigt) and FWHM calculations were carried out using the software *PDIndexer* (Seto *et al.*, 2010 ▸).

The performance of the newly designed Ge-SSDs with a CMOS-based preamplifier was explored on three different standard NIST materials, LaB_6_, CeO_2_ and Si (Fig. 9 ▸). The powders of these materials were slightly compressed in typical Kapton capillary tubes (0.8 mm internal diameter) also used at the powder diffraction beamline P02.1, DESY. Each capillary was placed on a mount positioned on the first-stage anvil in the center of the press. Results show a clear absence of a background in each diffraction pattern (Fig. 9 ▸), suggesting the scattered X-rays are sufficiently suppressed using the collimator-slit system and Pb detector shielding. Despite centimetre-thick shielding around the Ge-element of the detectors, some secondary Pb X-ray fluorescence is detected, as shown by the characteristic emission of Pb (*K*α and *K*β). The measurements for LaB_6_ and CeO_2_ show additional fluorescence lines from their respective heavy elements. Note that the diffraction pattern for LaB_6_ was obtained more recently using the new detector positioning system, whereas the other two patterns were obtained using the former temporary system.

During commissioning, the resolution limits of the Ge-SSD were determined to be 318 eV (at 59 keV) for ^241^Am and 467 eV (at 122 keV) for ^57^Co. Powder diffraction measurements are expected to offer slightly worse full width at half-maximum (FWHM) resolutions. For example, the peak for CeO_2_ at 125 keV has an FWHM of 581 eV [Fig. 9 ▸(*b*)], which is some 114 eV higher than γ-emission from ^57^Co. The peak for LaB_6_ at around 59 keV has an FWHM of 386 eV [Fig. 9 ▸(*a*)], which is only 68 eV higher compared with the γ-emission from ^241^Am. The new system appears to offer a slight improvement in energy resolution (FWHM) for LaB_6_ compared with CeO_2_ and Si obtained using the temporary system, although we compared different materials. Hence, under high pressure and temperature, the peak shape and FWHM resolution from sample diffraction are more likely influenced by microstructural-related effects, such as grain-size changes, texture and crystal defects (due to a deviatoric stress), use of samples with larger diameter, or any other effects that lead to peak-broadening.

Furthermore, CAESAR was carried out for the first time to explore whether this operation can be developed at the beamline. The main challenge is the need to simultaneously move two translation stages (*x*,*y*) and the rotation stage of a Ge-detector in order to rotate it around a virtual axis centered on the sample. At present, it appears a calibration is necessary over a large angular range to avoid missing the sample (*i.e.* without stage movement corrections, the gauge volume moves out of the sample during scanning). This calibration requires further optimization to minimize the sphere of confusion. However, it looks promising. Preliminary results are presented in Fig. 8 ▸. LaB_6_ powder inside a graphite cylinder (with an internal diameter of 1 mm) was scanned using detector 1. The acquisition time was 20 s for every 0.01° increment from 3° to 8°. Hence, in this case, the total acquisition time was approximately 167 min. The data obtained show the LaB_6_ reflections shift to lower energies with increasing detector position (2θ), while the characteristic X-ray emission from La and Pb (detector shielding) are visible as vertical straight lines (Fig. 8 ▸). For any given energy, an angle-dispersive XRD pattern can be extracted. The diffraction data is arguably spotty, which suggests the LaB_6_ grains are not homogeneously oriented, which can be corrected for using press rotation during each acquisition. The presence of graphite suggests some additional corrections are needed to ensure the gauge volume remains inside the sample while scanning. With these considerations, CAESAR operation is planned to be available for user beam time to enhance *in situ* crystallography and the study of amorphous materials in the LVP.

### 5.5. Quality of radiography data

The object resolution in radiography images from the X-ray microscope imaging system was also explored as well as determining if any difference in resolution using the 5× and 10× objective exists (combined with a GGG:Eu scintillator, 40 µm- and 20 µm-thick, respectively).

**Figure 10:**
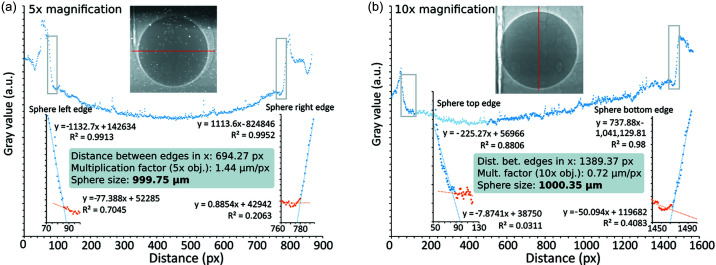
Radiographic imaging using the (*a*) 5× and (*b*) 10× microscope objectives on an extremely round metal sphere embedded in epoxy and evaluation of its diameter. The ‘bubbles’ visible in the images (particularly with 10× magnification) are air pockets in the epoxy tube. The red line in both images indicates the line profile used in the software *Fiji* (Schindelin *et al.*, 2012 ▸). Selected profile data points were used to fit linear functions, and the intercepts of the two functions at each end were adopted as the edges of the sphere. The distance between these two end points was calculated to obtain the sphere diameter for both 5× and 10× magnification, as indicated.

A near-perfect metal sphere (*d* = 1.0000 mm, roughness 0.010 µm) was embedded in epoxy and placed in the beam. The results of this resolution test are shown in Fig. 10 ▸. Unfortunately, the sphere quickly heated up by the wiggler beam causing the surrounding epoxy to melt around it within seconds. This can be observed in the images at both magnifications taken 0.1 s after opening the shutter. The first image offered the sharpest result although it cannot be ascertained whether the sphere had already started falling, blurring the edge of the sphere in the image. An intercept technique was used to offer sub-pixel resolution to obtain a result on the sphere diameter as 1000 µm with ∼1 µm uncertainty (Fig. 10 ▸). The determination of the object size in the image is therefore close to what was expected (*i.e.* 1.0000 mm), based on the camera field of view and image resolution (*i.e.* 3.7 mm/2560 pixel ≃ 1.44 µm pixel^−1^ for 5× magnification, 1.85 mm/2560 pixel ≃ 0.72 µm pixel^−1^ for 10× magnification). While sub-pixel image processing gives a decent result, the edges of the metal sphere imaged using both objectives are not sharp. This is fairly typical for far-field imaging at larger sample-to-detector distances using a highly divergent wiggler beam. Hence, the thickness of the scintillators does not likely play a critical role here in improving the image resolution, nor does the choice of microscope objective (5× or 10× magnification) matter much. However, it can be said that a secondary objective with a backup scintillator is worthwhile to keep during imaging experiments.

Note, the white halo around the sphere indicates that there is an additional phase contrast effect in the image besides absorption contrast. This can be useful for imaging interfaces and cracks in a cell assembly. Phase contrast occurs when imaging is carried out at larger distances (>1 m) and would be even more pronounced, for example, in the observation of different solids/liquids with the same effective atomic number *Z*_eff_, if the beam source was monochromatic with substantially more longitudinal coherence.

### 5.6. Deformation of NaCl at high pressure

A proof of concept experiment was performed on a 1.9 mm NaCl sample in the 8/5 assembly [Fig. 6 ▸(*b*)] compressed by third-stage SD anvils [Fig. 5 ▸(*c*)] to about 7 GPa and deformed at room temperature. Both Ge-SSDs were positioned at ∼7.5° in the 0° (D1) and 90° (D2) azimuthal positions, as described earlier. Due to a damaged collimator tube with a 50 µm gap, a tube with a 100 µm gap was used for the vertically inclined detector D1. The incident beam for ED-XRD was 100 µm × 150 µm, not quite a square beam to compensate for the difference in the collimator slit gaps (*i.e.* more beam towards D2 with a 50 µm vertical gap, than to D1 with a 100 µm horizontal gap). Regardless, the intensity of the diffracted X-rays in D1 is greater in the anvil gap than for D2 due to X-ray absorption from the cobalt binder in the SD anvil. The key results of the experiment are described next.

The application of a constant displacement rate at target *P* (and *T*) causes a deviatoric stress in the NaCl sample as indicated by a shift in peak positions in the ED-XRD patterns for D1 and D2 (Fig. S3). To quantify this shift, all resolvable peaks of NaCl were fitted in *PDIndexer* and the obtained lattice *d*-spacings of each *hkl* (see also Fig. S4) show an expected variation of *d*(*hkl*) as a function of the azimuth following equation (1). For this demonstration, the determination of *Q*(*hkl*) is sufficient to indicate there is a stress evolution in the sample resulting from the constant displacement of the hydraulic rams. Note, due to the lack of azimuthal detector coverage, there always remains some uncertainty about the true maximum and minimum of the sample stress, especially if the sample did not perfectly deform symmetrically under uniaxial compression.

**Figure 11:**
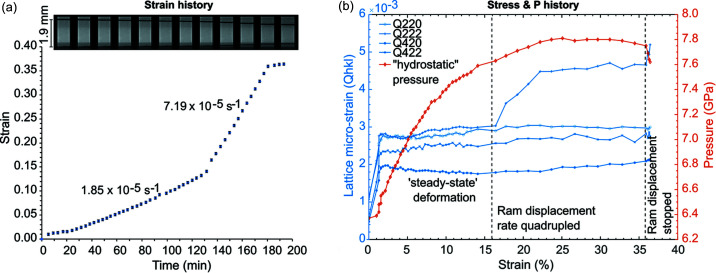
Deformation of NaCl at high pressure and room temperature. (*a*) Strain history was obtained by X-ray radiography. The black lines in the snapshot images are Pt metal foil disks between the sample and the alumina pistons and indicate the shortening (*i.e.* strain) in the sample at constant displacement rate of the hydraulic rams (1 and 2). The strain increments (blue points) were calculated as a function of time from each radiography image. The error in the strain estimation of each step is approximately ±0.0013 (symbol size). The data are fitted by linear functions to obtain the strain rate at each deformation step. (*b*) Diffraction data of NaCl (data points), obtained after each capture of a radiographic image are evaluated for *Q*(*hkl*), a representation of the lattice micro-strain (*i.e.* stress) for selected *hkl*, and plotted against strain. The pressure history during deformation is calculated from *d*_P_(*hkl*) using the equation of state in the work by Brown (1999 ▸). While random errors in Q(*hkl*) and *d*_P_(*hkl*) are expected to be low in the data, large systematic errors are expected (using only two Ge-SSDs) and are not evaluated here.

The macro-strain history, *i.e.* the shortening of the sample over time, is obtained by X-ray radiography using thin (25 µm) highly X-ray-absorbing Pt disks above and below the sample [Fig. 11 ▸(*a*)]. After initial compaction of other components in the cell assembly, the hard alumina pistons shorten the NaCl sample at a constant strain rate equivalent to the constant displacement rate of the hydraulic rams in the LVP. At a given point at around 130 min, the displacement rate was increased by a factor of four and hence the strain rate increased by a similar amount. The total strain accumulated in the sample was about 36%.

Using the information of *Q*(*hkl*) and the macro-strain, the stress–strain curve can be plotted for NaCl [Fig. 11 ▸(*b*)]. As shown, a sudden initial increase in ‘stress’ (elastic portion of the deformation) is followed by a long period of steady-state plastic deformation until the displacement rate (*i.e.* strain rate) increased in the second step. Note, *Q*(*hkl*) is not equal for all lattice planes, suggesting plastic anisotropy. The isostatic pressure obtained from *d*_P_(*hkl*) shows a significant increase of over 1 GPa during the first step of deformation and a more gradual increase during the second step of deformation until it plateaued, while the ‘stress’ further increased only for *Q*(222). Though this discrepancy cannot be explained with certainty, it is likely the sample fractured during the second step of deformation as the pressure and other stresses generally no longer increased despite a higher anvil displacement rate. Note, some materials may strengthen as a function of pressure. Hence to avoid such effects, the sample pressure should be kept constant by slightly reducing the oil pressure in the master ram to minimize the volume reduction of the pressure medium during deformation.

In summary, this experiment shows that the LVP, anvil-cell assembly and detectors can facilitate controlled *in situ* deformation experiments at high pressures (and high temperatures with an internal heater). Future modifications of the two Ge-SSD system and the future addition of a monochromator and area detector will further enhance these studies.

## 6. Conclusions and outlook

The high-energy wiggler beamline P61 offers a high-flux (filtered) white beam for *in situ* studies at two independently managed stations, engineering materials (HZ-Hereon at P61A) and extreme conditions research using the LVP (P61B). At P61B, the P61 wigglers and the high-count-rate Ge-detectors offer ED-XRD of excellent quality with short acquisition times of tens of seconds on the sample (*i.e.* >100 cps for peaks with the highest intensity), taking into consideration X-ray absorption of the cell assembly and pyrophyllite pressure-transmitting medium in the LVP. Furthermore, the detector positioning system supports two independently movable Ge-SSDs for capturing diffracted X-rays at multiple scattering angles simultaneously. Radiography is realized using an X-ray microscope equipped with a thin GGG:Eu scintillator on two magnifying objectives and additional shielding to protect the objectives and sCMOS camera.

The six-ram LVP, Aster-15 installed at P61B, offers multiple modes of pressure generation and a large chamber for compressing a variety of anvil-cell assembly designs for *in situ* X-ray studies at high pressure and high temperature. Furthermore, the precise alignment and control of the six hydraulic rams permits ultra-high-pressure generation to over 50 GPa and simultaneous high temperatures (>3000 K) with user-specialized assemblies and anvils. The press control, AC and DC heating, movements of the press stages and slits, as well as all the data acquisition functions are programmed with user-friendly graphical user interfaces (GUIs), designed in-house. Advanced experiments may be complemented with additional instrumentation, such as the AE detection system to measure the cracking mechanisms and the UI system to measure acoustic wave velocities in the sample. P61B also offers a benchtop diffractometer for additional XRD measurements (particularly for user experiments not receiving synchrotron X-rays). Finally, the installation of a monochromator is anticipated to enhance capabilities for *in situ* studies of rock deformation and crystallography using AD-XRD. This, and other ideas (such as CAESAR operation and the addition of a Paris–Edinburgh press from the PETRA III Extreme Conditions Beamline, P02.2). are on the list for future development at P61B in the next few years, with the consideration and requirement of the necessary financial support and available commissioning time.

## . Related literature

The following references, not cited in the main body of the paper, have been cited in the supporting information: Cottar *et al.* (2004 ▸); Wojdyr (2010 ▸).

## Supplementary Material

Data acquisition and processing. Figures S1 to S4, Table S1. DOI: 10.1107/S1600577522001047/ju5040sup1.pdf

## Figures and Tables

**Table 1:** Machine parameters of the PETRA III storage ring, including extensions (Bacher *et al.*, 2007 ▸) and the parameters of the ten damping wigglers (Drube *et al.*, 2016 ▸)

Machine parameters		Insertion device parameters P61		Beam characteristics at P61B (EH2)	
Energy	6 GeV	Device	10× wigglers	Energy range	30–160 keV†
Circumference	2304 m	Minimum magnetic gap	24 mm	Maximum beam	2.2 mm (h) × 1.7 mm (v)
Harmonic‡	3840	Period length λ_U_	200 mm	In-vacuum	10 mm densimet
HF	500 MHz	Device length *L*	10 × 4 m	Slits thickness	(W-alloy) blades
Horizontal	1.2 nm rad	Distance between IDs	2 m	Minimum aperture (µm)	10 × 10
emittance		Number of periods	10 × 19	Exit window	CVD diamond (water-cooled)
Coupling factor	1%	Peak field *B*_0_	1.52 T		
Beam current	120 mA (m.b.)§	Deflection parameter *K*_max_	28.4		
	100 mA (40-b.)¶	First harmonic *E*1	35.8 keV (*E*_c_)		
No. of bunches	960 or 40	Total power *P*_tot_	10 × 21 kW		
β*x*	20.12 m	On-axis power density	10 × 44 kW mrad^−2^		
β*y*	2.36 m	Power in 1 mm × 1 mm at 40 m	121 W		

† Calculated using *OASYS REF* (Rebuffi & Sanchez del Rio, 2017 ▸).

‡ Energy range binned with 25 parts per 1 keV (4096 channels (Quantum detectors Xspress 3 mini digital analyser).

§ m.b. = multi-bunch brightness mode.

¶ 40-b. = 40-bunch timing mode.
