# Supplementary material for: Extreme conditions research using the large-volume press at the P61B endstation, PETRA III
Source: J Synchrotron Radiat. 2022 Feb 22;29(Pt 2):409–23. doi: 10.1107/S1600577522001047 (PMC8900846; doi:10.1107/S1600577522001047)
Supplement: Supplementary file 1 [file s-29-00409-sup1.pdf]

JOURNAL OF SYNCHROTRON RADIATION

Volume 29 (2022)

Supporting information for article:

Extreme conditions research using the large volume press at the P61B endstation, PETRA III

Robert Farla, Shrikant Bhat, Stefan Sonntag, Artem Chanyshev, Shuailing Ma, Takayuki Ishii, Zhaodong Liu, Adrien Néri, Norimasa Nishiyama, Guilherme Abreu Faria, Thomas Wroblewski, Horst Schulte-Schrepping, Wolfgang Drube, Oliver Seeck and Tomoo Katsura

S1. Data acquisition and processing

Custom in-house software is available to acquire X-ray diffraction data using 1 or 2 Ge-SSD and imaging data using the X-ray microscope. The beamline controls are scripted with a graphical user interface using python and Qt, calling the operation of the Tango servers for the various devices. Users have access to the stage movements of the LVP and slit system to shape the beam for diffraction or imaging. Pre-sets can be set up during an experiment to move to various locations in the cell assembly under pressure into the beam (e.g. sample and pressure marker locations). In order to prevent user error and unintended damage, some functions are not accessible to the user, such as individual stage movement of the detectors to avoid collisions between the detector components and the LVP.

Data acquisition for ED-XRD is implemented using a custom graphical user interface as follows. Data are saved in the hdf5 (nxs) file format and can be acquired simultaneously using 2 Ge-SSDs. There is no limit on the acquisition time, although the maximum time per frame is 50s. So, if 100 s of acquisition is needed, two frames of 50 s are used. For each acquisition, several options can be included in the file name such as, a counter, a user comment, duration, detector positions, press-stage positions, *etc*. Additionally, a log file is saved with the same file name containing a time stamp and all the information of the state of the experiment (pressures and positions of the LVP rams, the furnace parameters if DC heating is used, motor positions of the slits and press stages, list of detected peaks and their intensities, and beam status with current and energy information). A final third option available for each acquisition is a direct conversion of the diffraction data in hdf5 nxs file data format to readable csv format saved separately in the ‘processed’ directory of the beamline gpfs filesystem.

Data acquisition for radiography imaging is similarly implemented using a custom graphical user interface. Data may be acquired in hdf5 format or uncompressed tif format. When huge quantities of frames are to be captured, hdf5 container format is preferred. The following available functions are separated for data saving mode and live view mode. In data saving mode, an image or a series of images can be saved with a chosen acquisition time and delay, typically 0 s. For live view mode, only acquisition time can be modified before starting the acquisition. For both modes, a region of interest (ROI) can be implemented in order to improve the refresh rate of the camera and reject any portion of the image (e.g. where no beam is available). The software additionally features a toggle switch for 5x and 10x magnification, as well as the option for incremental z-stage image acquisition if the entire length of the assembly or a sample larger than the beam in the vertical direction, is to be imaged.

Typically, during data acquisition, immediate feedback is required for the experiment. For instance, direct estimation of the pressure and temperature conditions in the cell assembly is needed, as well as the analysis of diffraction patterns for the occurrence of a phase transition or phase nucleation. To

meet these needs, the control hutch is equipped with extra PCs for data processing with various freely available software installed. The main software used for ED-XRD data processing is called *PDIndexer* (Seto *et al.*, 2010), modified by the author for data processing at P61B. The programme reads the hdf5 (nxs) file format written by the Tango device for the Xspress digital analyser using libraries from the HDF Group (https://www.hdfgroup.org/). Upon loading data files, a dialog opens with the pre-entered information of the channel-energy calibrations for the detectors and the calibrated 2θ angle of the detector. The software can be used to estimate pressure based on a range of materials with established equations of state (such as MgO and NaCl), and peak fitting is possible from crystal data of any material in the list or imported from a cif structure file. For more information about the software, please visit the author's software homepage (http://pmsl.planet.sci.kobe-u.ac.jp/~seto/).

P61B offers additional python-based software tools to perform the following functions: 1) data conversion tool between hdf5 (nxs) format to csv, 2) precise determination of detector 2θ angle from MgO or NaCl peak positions manually fitted by other software (*Fityk*, Wojdyr, 2010), 3) ram oil pressure to sample pressure estimation for MA6-8 and MA6-6 compression modes for a variety of commonly used assemblies (cell size and anvil truncation), 4) simultaneous estimation of pressure and temperature from the experimental lattice constants (or volume) combined with established equation of states of commonly used materials (Fig. S3). This application uses various functions of the freely available *Burnman* tool (Cottaar *et al.*, 2014). Lastly, for radiography, rapid feedback on sample length changes may be needed. The use of the freely available software *ImageJ* (or *Fiji*, a plugin supported version) is highly recommended (Schindelin *et al.*, 2012).

Table S1 LVP and detector system specifications.

**mavo press LPQ6-1500-100**	6 indep. controlled rams
Maximum load	15 MN – 5 MN/axis
Ram position control	1 µm step – 100 mm stroke
Oil pressure control	0.5 bar – 620 bar/ram
Anisotropic compression	Axial symmetric, triaxial
5-axis stage	x,y1,y2,z (± 100 mm), ϕ ± 11.5° (with detectors)
Combined weight	ca. 45 ton
**Detector Positioning System**	DESY-designed, Kohzu-built
D1 & D2 full-step resolution / repeatability	
X-translation	~7.0 µm / 0.2 µm
Y-translation	~6.5 µm / 0.2 µm
Z-translation	~1.0 µm / 0.1 µm
Z-rotation	~0.0025° / 0.0003°
Y-rotation (unit 1 only)	~0.0014° / 0.0003°
Collimator slit gap (mm)*	0.03, 0.05, 0.1, 0.2
Receiving slit gap (mm)#	0.05, 0.1, 0.2, 0.5, 1.0, 2.0
Horz. detector positions	1xGe: min 3° - max 20°
	2xGe: min 3° - max 10°
Horz. & vert. positions	Ge_{vert}: min 3.0°$ - max 23°
	Ge_{horz}: min 6.5° - max 10°
**Ge-SSD (2x)**	Mirion (Canberra)
Preamplifier type	CMOS, 3.5 mV/keV gain
Ge-element	100 mm^2, 10 mm thickness
Window	0.5 mm Al
Cryostat	Electric, Cryopulse 5 Plus,
	constant temperature: -190 °C
Digital analyser	2-Ch 4k Xspress3 mini (Quantum Detectors)
**White beam X-ray microscope**	Optique Peter - PCO.edge
Magnification	5x/0.14 LWD, 10x/0.28 LWD, [20x/0.42 LWD)]
Scintillator (2x objectives)	GGG:Eu (10 to 40 µm) & LuAG:Ce (20, 40 µm)
Mirror	Glassy carbon
Camera	sCMOS PCO.Edge 5.5 MP
Hor. Object field (mm)	3.7 (5x), 1.85 (10x), 0.92 (20x)
Vert. Object field (mm)	3.12 (5x), 1.56 (10x), 0.78 (20x)
Diag. field of view (mm)	4.84 (5x), 2.42 (10x), 1.21 (20x)
Object H&V pixel size (µm)†	1.44 (5x), 0.72 (10x), 0.36 (20x)
+/- Depth of focus (µm)	19 (5x), 4.8 (10x), 2.0 (20x)
Quantum efficiency	60% at peak wavelength (600nm)
Resolution / frame rate	2560 x 2160 @ 100 fps, over 1000 fps for a ROI
Shutter	Rolling and true global

* smallest gap, 1 mm in other direction; # gap size for x,y directions can be chosen independently;

$ lower angles currently inaccessible due to collision of direct beam with detector electronics; † theoretical optical resolution limit (not actual image resolution)

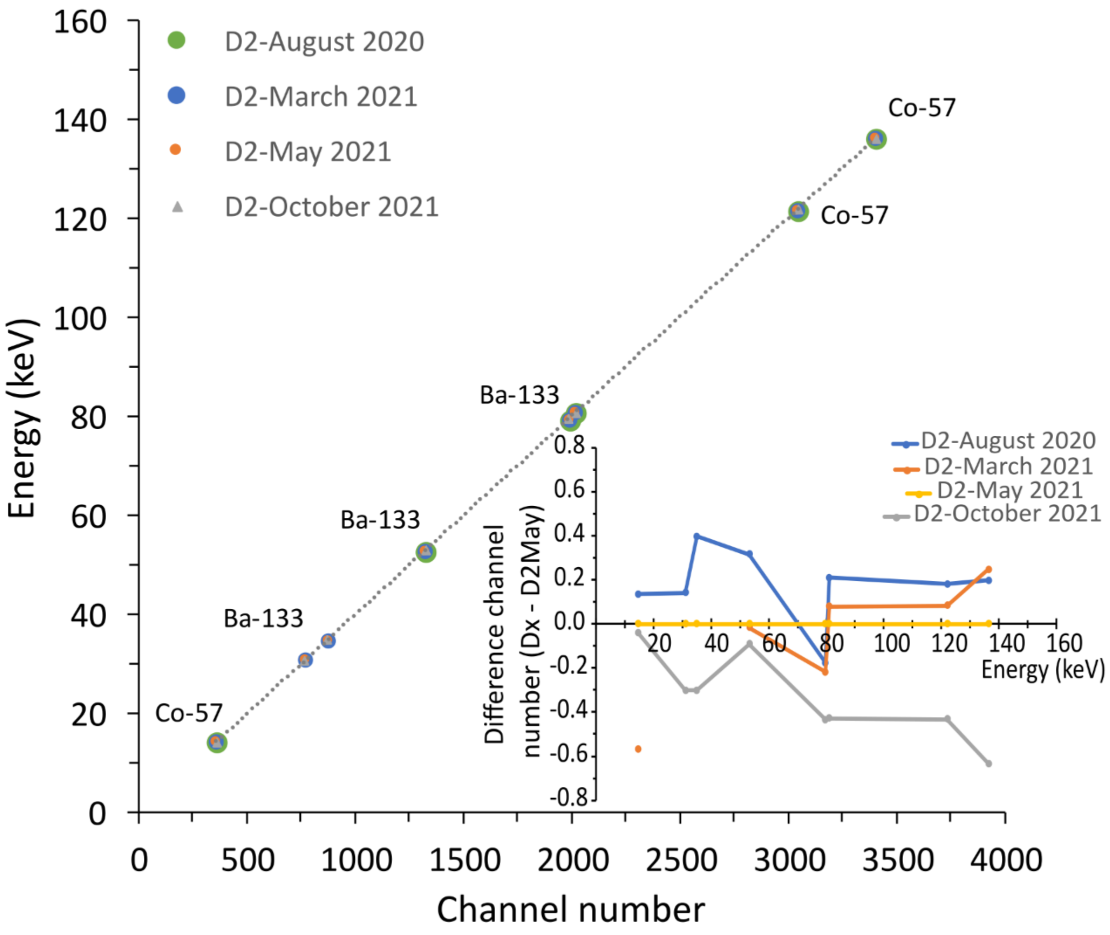

Figure S1 Channel-energy calibrations of the most-used detector D2. The calibrations were carried out between August 2020 and October 2021 using the radionuclides ^{133}Ba and ^{57}Co. A quadratic equation is used for fitting the data even though the relationship between Energy and Channel number is essentially linear (user choice). The inset shows the deviations among the three calibrations for D2. To date, the calibrations agree very well and remained very consistent.

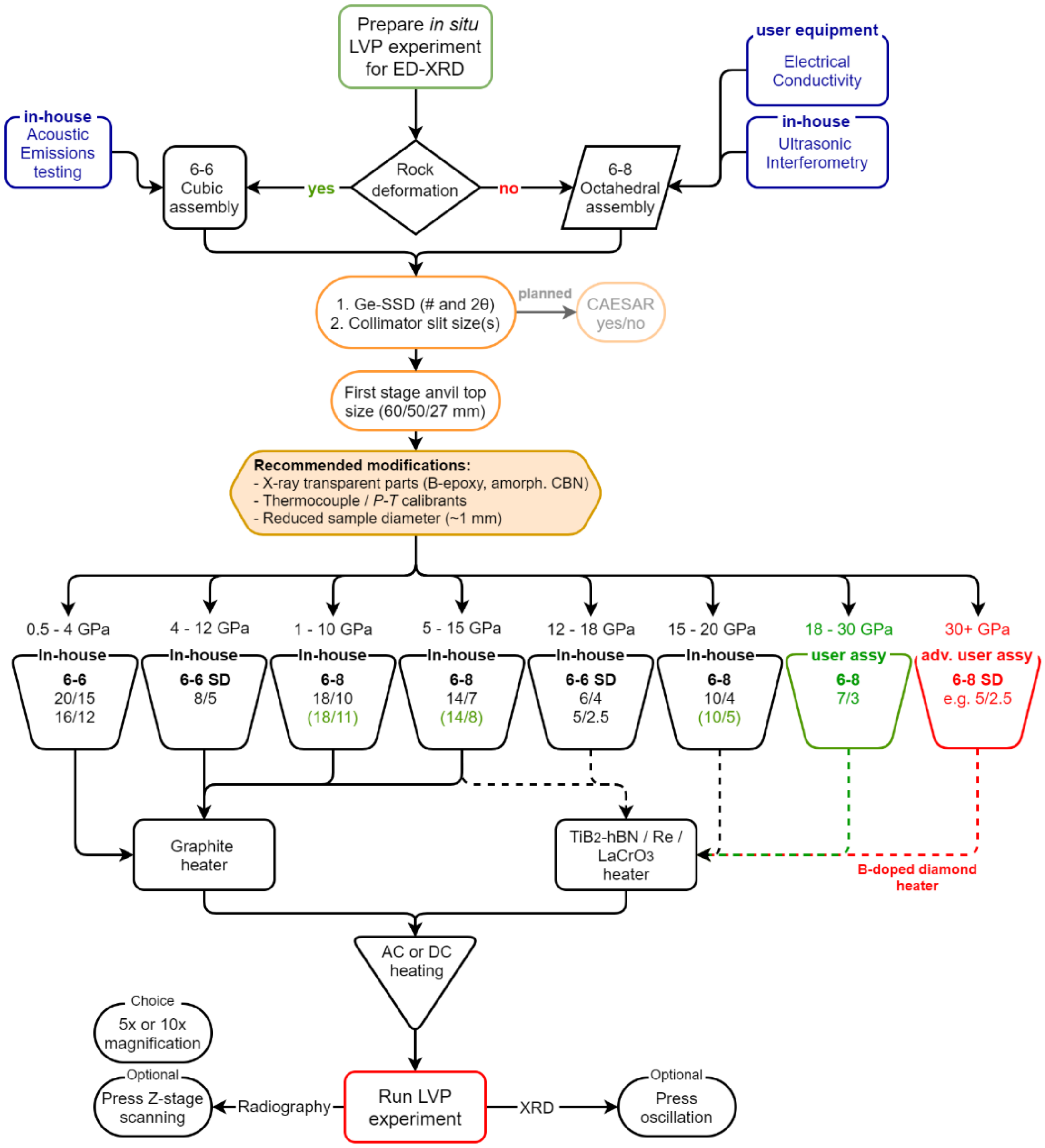

Figure S2 A user guide for *in situ* LVP experiments at P61B.

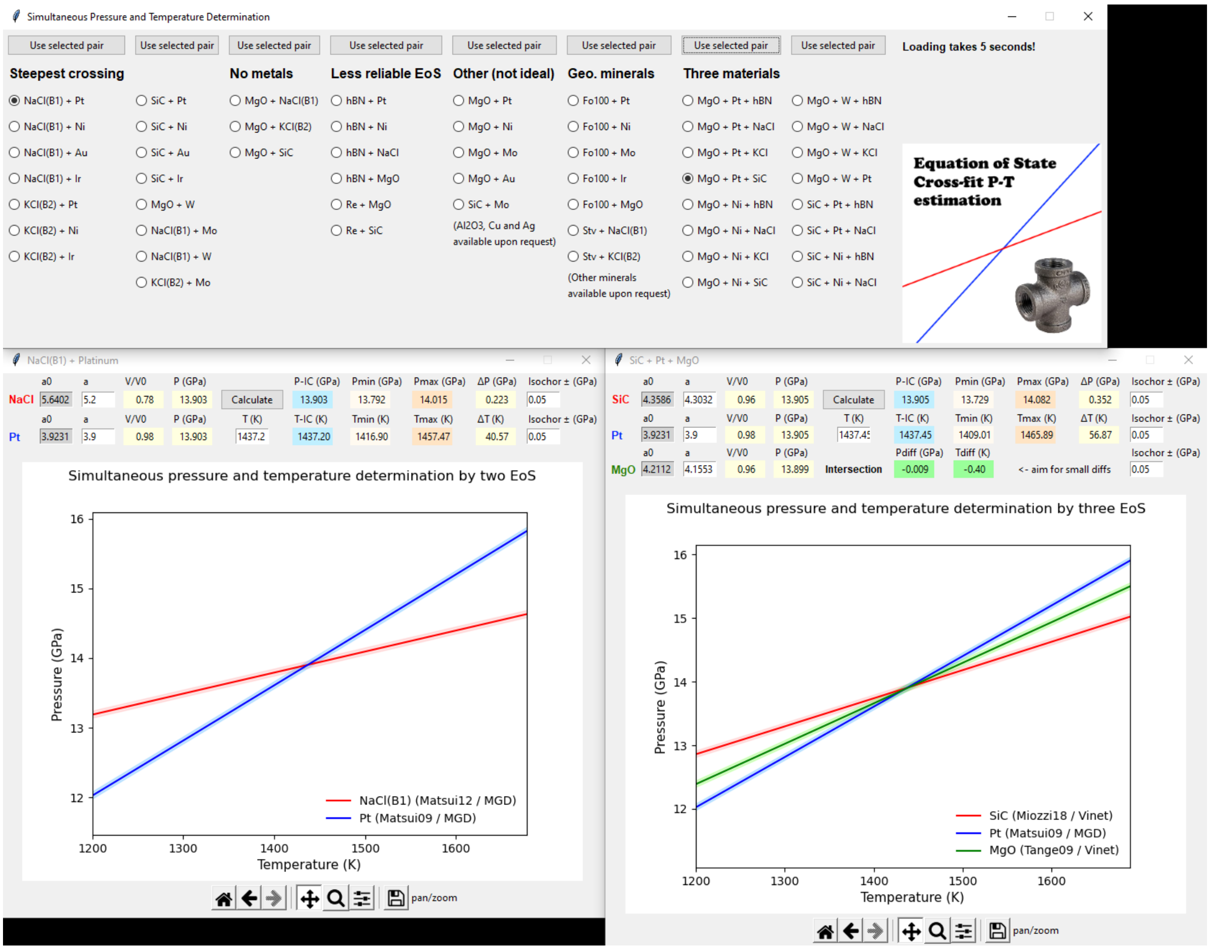

Figure S3 Screenshot of the in-house, python-based application *Cross-fit* for estimating pressure and temperature conditions simultaneously *in situ* by application of the respective equations of state of two materials in a mixture. The only required inputs are the lattice constants *a* (or volume *V*) of 2 or 3 phases. Optionally, a temperature value can be entered to calculate the pressure from the equation of state of a phase with given lattice constant. For any case, the lattice constant *a0* of a material at ambient conditions can be changed if the default value is not desired. The uncertainty on the pressure and temperature determination (min and max values) is semi-quantitatively explored by applying a shift in the positions of the isochors in pressure (default: ± 0.05 GPa). Ideally, the errors in the lattice constants (or volume) are taken for materials at given P and T conditions. These errors arise from the uncertainty in the peak positions fitted by a model function (e.g. a Voigt profile). For simplicity, this was not implemented. If interested in the application, please contact the corresponding author.

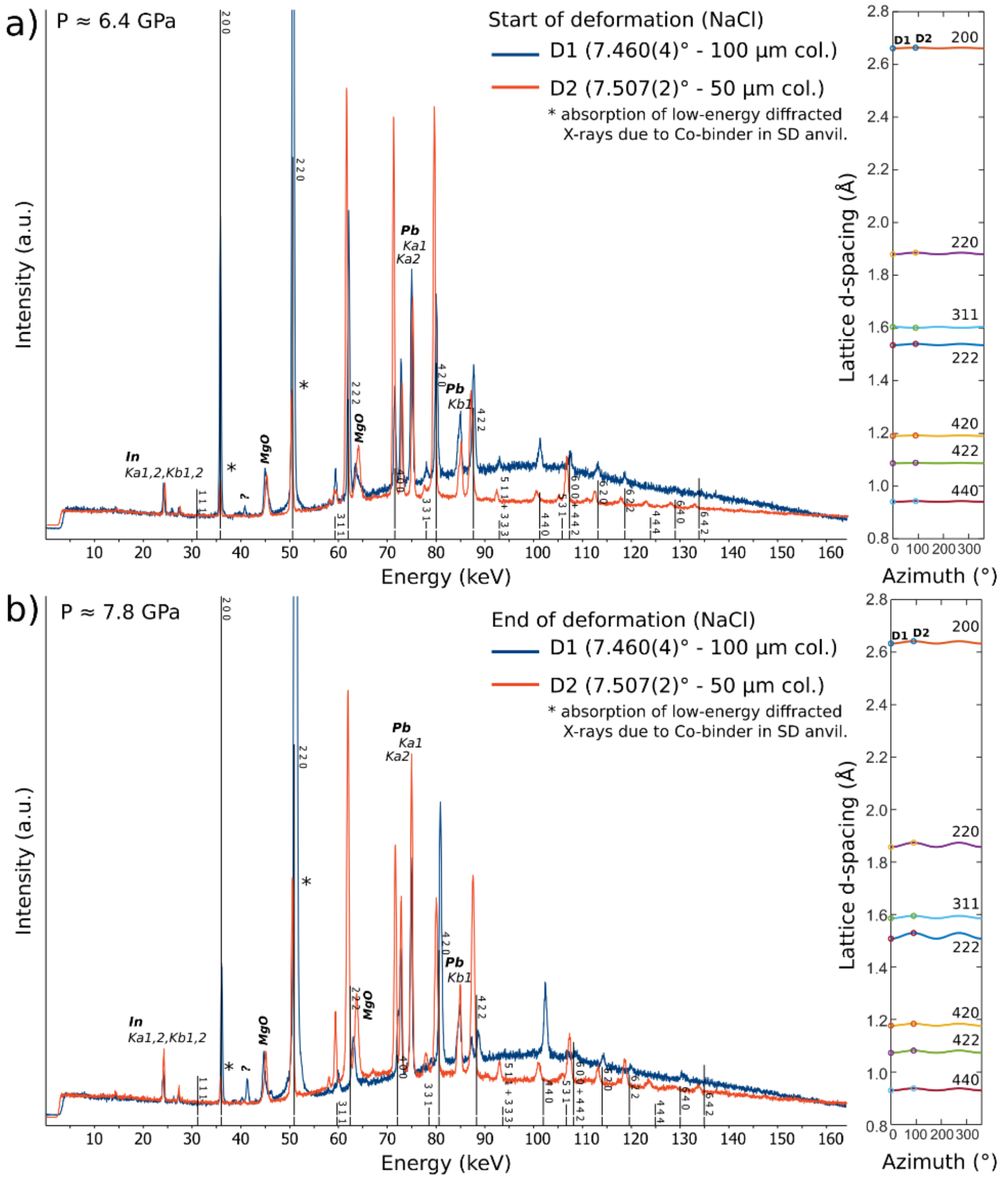

Figure S4 Selected diffraction patterns obtained from NaCl using 2 Ge-SSD simultaneously a) before the start and b) at the end of deformation. The conditions of the acquisitions are as indicated. Peak fitting was carried out using the software PDIndexer (Seto *et al.*, 2010). The positions of the peaks (*i.e. d*-spacing) in the diffraction patterns, were plotted against the azimuthal positions of the detectors (0° and 90°, respectively). The expected variations of *d*-spacing with azimuth are also shown by the wavy colored lines calculated using Equation 1 (see text). The hydrostatic pressures are calculated from $d_P(hkl)$ (Eqn. 1).

References

Cottaar, S., Heister, T., Rose, I. & Unterborn, C. (2014). *Geochem. Geophys. Geosystems*. **15**, 1164–1179.

Schindelin, J., Arganda-Carreras, I., Frise, E., Kaynig, V., Longair, M., Pietzsch, T., Preibisch, S., Rueden, C., Saalfeld, S., Schmid, B., Tinevez, J.-Y., White, D. J., Hartenstein, V., Eliceiri, K., Tomancak, P. & Cardona, A. (2012). *Nat. Methods*. **9**, 676–682.

Seto, 瀬戸, 大輔浜根, 隆哉永井 & 永吉佐多 (2010). 高圧力の科学と技術. **20**, 269–276.

Wojdyr, M. (2010). *J. Appl. Crystallogr*. **43**, 1126–1128.
